# Blood-Letting

**Published:** 1873-09

**Authors:** 


### BLOOD-LETTING.

The following, from a very recent number of the *British Medical Journal*, has seemed to us to be of sufficient importance to be worthy of a prominent place in our issue :

"We gladly welcome such researches as have been lately published by Bauer in the *Zeitschrift fur Bioligie*, whose experiments were made for the purpose of discovering what changes occur in the metamorphosis of albumen and fat in the body after venesection. One would expect *a priori* that the withdrawal of a quantity of blood, by merely lessening the the amount of albuminous substances in the body, would be followed by a diminution in the daily metamorphosis. So far from this being the case, however, Bauer finds that the quantity of albuminous bodies decomposed daily in the organism is invariably increased by blood-letting, and the excretion of the urea which is formed by their decomposition is consequently augmented. Notwithstanding this, however, much less oxygen is consumed than before, so that the substances into which albumen splits up cannot undergo such perfect combustion as before the venesection. One may readily understand this, for it is the red blood-corpuscles which carry oxygen throughout the body ; and when their number is lessened by blood-letting, combustion in the tissues can hardly go on so rapidly as before. The decomposition of albumen in the body is altogether independent of the oxygen consumed in it. Now, fat is one of the substances furnished by this decomposition ; and, as its formation is increased and its combustion lessened, to say nothing of the fat taken as food, it must accumulate in the body ; and this it actually does, as was well known to the phlebotomists of former days, whose writings contain many a record of cases where patients became enormously fat after copious blood-lettings. The same thing is seen in a milder form almost every day in the case of chlorotic girls, on whose bodies fat becomes deposited, because they have too few red blood-corpucles to carry it to the oxygen required for its combustion. In many localities, too, cattle-breeders have become acquainted with the fact, and they increase the quantity of fat formed by their animals either in the shape of butter yielded by their milch-cows, or accumulated on the bodies of the oxen they wish to fatten by bleeding them from time to time.

"In diseases such as pneumonia, blood may be drawn for the purpose of diminishing the temperature or of lessening dyspnœa. It may be supposed that Bauer's experiments support the use of blood-letting for the former purpose ; but this is not the case, for the fall of temperature which blood-letting produces takes place immediately, and is soon over, while combustion does not diminish till some hours have elapsed. The fall of temperature is, therefore, due to another cause,—viz.: the relaxation of the superficial vessels allowing the blood to cool more readily ; but temperature can be reduced much more quickly and effectively by the cold douche or wet sheet: and increased transformation of albumen, with diminished oxydation, is not unlikely to lead to a fatty degeneration of important organs.

"The relief which venesection generally affords in dyspnœa probably depends in part on its effect in cooling the body, for Fick and Goldstein have shown that increased temperature of the blood is sufficient to produce dyspnœa. This is a real benefit; but it would be attained as well by the use of a wet sheet, and a second cause of a subjective relief after blood-letting is by no means so desirable. The loss of blood, according to Traube, diminishes the irritability of the medulla oblongata, which is the center of innervation for the respiratory muscles, and, by thus producing a sort of narcosis in this part of the nervous system, deprives it of its power to appreciate rightly the respiratory wants of the body. A third way in which it may prove useful in dyspnœa is by lessening the resistance, which hinders the right ventricle from emptying itself completely, and, by thus facilitatating the circulation in the lungs, may assist respiration. The author considers that this condition will only last until as much fluid has been absorbed into the vessels from the tissues as will restore the blood to its former volume ; but of this we are not quite sure.

"Lastly, he says it is not to be denied that the danger arising from serous exudations in important organs, as well as congestion of the brain or lungs, may be temporarily averted by general blood-letting, in consequence of the absorption of fluid into the vessels which it occasions. This observation has been too often made to leave any doubt upon the subjeet; but as often has it been noticed that the danger of these conditions recurring increased at every venesection. Œdema is to a great extent dependent on weakness of the vaso-motor nerves (see this Journal, June 15, 1872, p. 644); and, this weakness being increased by blood-letting, the œdema is more likely to occur. Bauer, however, gives a case which shows most strikingly the immediate benefit produced by blood-letting in a case of œdema of the lungs; and we are inclined to think that the weakness of the vaso-motor nerves which might lead to a recurrence of the œdema might be successfully combated by a vaso-motor tonic, such as digitalis. While this shows that immense benefit may be derived from its use in proper cases, the effects which it produces on the tissue-change in the organism teach us that it is not a remedy to be thoughtlessly used, but one which must be employed only after due consideration and with watchful care.

